# Lane‐Hamilton syndrome

**DOI:** 10.1002/rcr2.1188

**Published:** 2023-07-04

**Authors:** Audrey K. Grech, Christiaan Yu

**Affiliations:** ^1^ Department of Respiratory Medicine Alfred Health Melbourne Victoria Australia; ^2^ Central Clinical School Monash University Melbourne Victoria Australia

**Keywords:** celiac disease, hemoptysis, idiopathic pulmonary hemosiderosis, immunosuppression, Lane‐Hamilton syndrome

## Abstract

The co‐existence of idiopathic hemosiderosis and celiac disease is Lane‐Hamilton Syndrome. This is a rare condition with only a few dozen cases reported to date. Its clinical presentation typically involves hemoptysis that can be life‐threatening in the acute phase. We report the uncommon case of the development of idiopathic pulmonary hemosiderosis almost a decade after the diagnosis of celiac disease. Delayed diagnosis led to recurrent episodes of large volume hemoptysis despite immunosuppressive therapy due to ongoing ingestion of gluten. High doses of glucocorticoids accompanied by a cell cycle inhibitor mycophenolate mofetil were required for treatment. A strict gluten free diet is vital to control the disease. We highlight the importance of identifying this syndrome and definitive treatment, including avoidance of dietary triggers in addition to conventional immunosuppressive therapy.

## Graphical abstract

We present a previously undescribed occurrence of Lane‐Hamilton syndrome, where there was a notable latency between abdominal and pulmonary manifestations. This resulted in a diagnostic delay and disease control was ultimately achieved through immunosuppression and a gluten free diet, highlighting the importance in achieving a timely diagnosis.

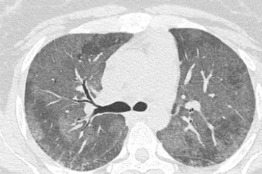

## INTRODUCTION

Lane‐Hamilton syndrome is a rare condition defined by the co‐existence of idiopathic pulmonary hemosiderosis (IPH) and celiac disease. Since it was first described in 1971, there have been less than a hundred cases reported worldwide.^1^, ^2^ Recognizing this syndrome is of utmost importance as a delayed diagnosis can lead to repeated episodes of large volume haemoptysis which carries significant mortality.^3^

We present a previously undescribed occurrence of Lane‐Hamilton syndrome, where there was a notable latency between abdominal and pulmonary manifestations. This resulted in a diagnostic delay and disease control was ultimately achieved through immunosuppression and a gluten free diet, highlighting the importance of achieving a timely diagnosis.

## CASE REPORT

A 29‐year‐old woman with a past history of celiac disease presents with recurrent large‐volume hemoptysis to the emergency department.

At the age of 20, she was diagnosed with celiac disease following investigations for lethargy and iron deficiency anaemia. Endoscopic biopsies revealed lymphocytic gastritis and small bowel severe villous atrophy with increased intraepithelial lymphocytosis. Anti‐gliadin and anti‐tissue transglutaminase antibodies were negative. Genetic testing showed that she was HLA‐DQ2 homozygous positive. She did not have any respiratory symptoms, and her chest imaging did not show any abnormalities. Her condition had been stable on a gluten‐free diet.

In the past year, following a reversion to a gluten‐containing diet, she had two similar episodes of hemoptysis. She was treated with an 8‐week course of oral corticosteroids each time. Her diet was not altered. Between episodes, she had a flexible bronchoscopy that appeared unremarkable. Bacterial and fungal culture was also negative. She proceeded to have a right video‐assisted thoracic surgery upper lobe wedge biopsy which demonstrated haemosiderin‐laden macrophages and lymphocytic bronchiolitis consistent with IPH.

**FIGURE 1:**
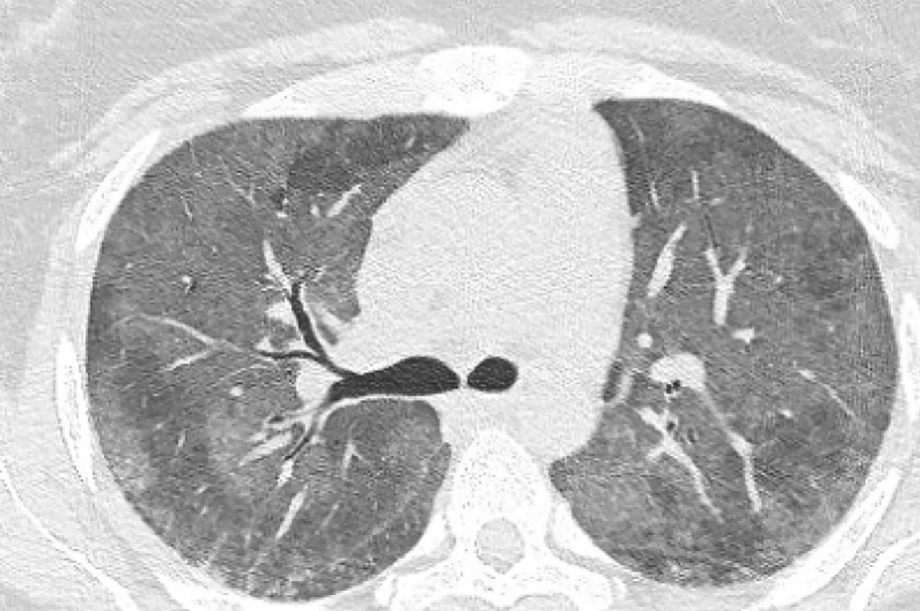
Computed tomography (CT) chest showing bilateral ground glass infiltrates in the upper lobes of the lung. Differing attenuation in various regions results in a mosaic attenuation.

During this presentation, she experienced progressive dyspnoea, pleuritic chest and abdominal pain. She was not on any anti‐platelet or anti‐coagulation therapy. On initial examination, her heart rate was 130 beats per minutes (regular), blood pressure 160/95, respiratory rate 24 breaths per minute and oxygen saturation was 88% on room air. Clinical examination revealed crepitations throughout both lung fields. Computed tomography pulmonary angiogram showed bilateral ground glass infiltrates with mild interlobular septal thickening (Figure 1). Of note, there was no pulmonary embolus detected. Nasopharyngeal aspirate did not detect any viruses. There was no evidence of a concomitant vasculitis as her anti‐nuclear antibodies (ANA) and anti‐glomerular basement membrane (anti‐GBM) were negative. Her c‐ antineutrophil cytoplasmic antibodies (ANCA) was weakly positive but MPO and PR3 were negative. Complement 3 and 4, rheumatoid factor, C‐reactive protein and creatine kinase were within normal limits. Kidney function was within normal limits, estimated glomerular filtration rate (eGFR) >90, Cr 60 and 11 × 10^6^/L (normal ≤10 × 10^6^) erythrocytes with no active sediment found on urine microscopy. She was anaemic with a haemoglobin of 80 g/L, platelets were 296 × 10^9^/L and coagulation studies were unremarkable.

In the first 24 h, she received 2 units of packed red blood cells. In addition, she was given a 3 day pulse of methylprednisolone (1 g daily) and commenced on mycophenolate mofetil 750 mg. Oral prednisolone was commenced at 1 mg/kg following the pulse and slowly weaned off over the next 3 months. A comprehensive dietary review discovered the presence of gluten in her daily intake and was subsequently removed from her diet.

Twelve months following this episode, the patient has progressed well and has no further episodes of significant hemoptysis. With the help of a dietician, she remains on a gluten‐free diet. Repeat gastroscopy demonstrated complete resolution of the villous atrophy and intraepithelial lymphocytosis. She remains on the single immunosuppressive agent of mycophenolate mofetil.

## DISCUSSION

Lane‐Hamilton syndrome, the co‐existence of IPH and celiac disease, is a rare syndrome that confers a mortality of 14% during the acute phase. The exact incidence of this condition is unclear, and there are only case reports, with a recent review identifying only 80 cases over the past 30 years.^2^ The paucity of data in this field limits awareness and treatment experience.

IPH is characterized by repeated episodes of diffuse alveolar haemorrhage and the absence of an apparent underlying cause. The prognosis and survival time in IPH varies widely. Life threatening hemoptysis is the most common cause of morbidity and carries a modest mortality during an acute exacerbation.

Celiac disease, one of the most common food‐related disorders, is an immune‐mediated enteropathy against dietary gluten found in wheat, rye and barley. Upon ingestion in susceptible individuals, prolamines found in gluten triggers the disassembly of intestinal cells and induction of pro‐inflammatory cytokines in the small intestine. Accounting for a third of the total genetic risk, is the presence of gene encoding for major histocompatibility complex (MHC) class II, proteins including HLA DQ2 and HLA DQ 8.

Although Celiac disease and IPH are both immunologically mediated, the pathogenetic link between them is not known. In Lane Hamilton syndrome, similar to IPH, systemic corticosteroids are the mainstay of treatment. Long‐term steroids are not well tolerated, and early withdrawal of treatment may lead to disease recurrence. A variety of other immunosuppressive drugs, such as hydroxychloroquine, azathioprine, cyclophosphamide and mycophenolate mofetil have been trialled as adjunctive therapy and may be indicated based on comorbidities.^4^ It is vital to identify any gluten in the patient's diet as this can lead to breakthrough exacerbations despite a dampened immune state. In Lane‐Hamilton syndrome, lifelong adherence to a strict gluten‐free diet is the cornerstone of therapy.

Finally, there has been no published data on the observed interval between gastrointestinal and pulmonary manifestation. It is plausible that ongoing exposure to gluten may progress and accelerate the disease.

We highlight the challenges in the diagnosis of Lane‐Hamilton syndrome and raise awareness of the need to consider this condition as a differential in those with IPH. There may be a temporal delay between diagnosis of Celiac disease to the first episode of hemoptysis. Considering the implications on treatment, it may be prudent to screen for Celiac disease and conduct a dietary history, particularly when no clear cause for hemoptysis is found. Aggressive immunosuppression remains the mainstay of life‐threatening episodes of large volume hemoptysis, while a steroid sparing agent such as mycophenolate mofetil can be considered as longer term therapy.

## AUTHOR CONTRIBUTIONS

**Audrey K. Grech**: Writing and final edit. **Christiaan Yu**: Writing and final edit.

## CONFLICT OF INTEREST STATEMENT

None declared.

## ETHICS STATEMENT

The authors declare that appropriate written informed consent was obtained for the publication of this manuscript and accompanying images.

## Data Availability

Data sharing not applicable to this article as no datasets were generated or analysed during the current study.
